# Metabolic orchestration of drug-tolerant persister cells in cancer

**DOI:** 10.1093/lifemedi/lnae040

**Published:** 2024-12-07

**Authors:** Meng Nie, Zeping Hu

**Affiliations:** School of Pharmaceutical Sciences, Tsinghua-Peking Joint Center for Life Sciences, Tsinghua University, Beijing 100084, China; School of Public Health, Capital Medical University, Beijing 100069, China; School of Pharmaceutical Sciences, Tsinghua-Peking Joint Center for Life Sciences, Tsinghua University, Beijing 100084, China

Despite significant advancement in developing effective anticancer therapies, drug resistance remains a major clinical obstacle to achieving durable cures. This has sparked extensive research efforts into the underlying mechanisms of acquired resistance to cancer treatment, unveiling genetic alterations, dysregulated signaling pathways, and intricate interactions within the tumor microenvironments. However, the highly complex and heterogeneous nature of drug-resistant cancer cells makes them hard to eliminate. Hence, very few combination therapies designed to combat resistance have made their way into routine clinical care.

In a seminal study published in 2010, Sharma et al. identified the drug-tolerant persister cells (DTPs), as a rare slow-cycling subpopulation of cancer cells, can acquire a reversible quiescent state, allowing them to evade initial drug treatment and eventually evolve to irreversible drug resistance [1]. Since then, a decade of research has detected DTPs across a wide range of cancer types under diverse chemotherapies and targeted agents, which is also referred to as minimal residual disease acting as a key driver of tumor relapse in clinical settings. Therefore, elucidating the biological characteristics and therapeutic vulnerabilities of DTPs is crucial for tackling drug resistance and achieving long-lasting clinical responses for cancer patients. Recently, mounting evidence has shed light on the regulatory mechanisms contributing to the emergence of cancer persistence, including epigenetic, transcriptional, and metabolic reprogramming as well as complex interactions between tumor cells and microenvironment. While epigenetic and transcriptional remodeling in DTPs have been extensively reported, the transitory nature of DTPs phenotype underscores the importance of both cell-autonomous and non-autonomous metabolic adaptions [2]. Indeed, the metabolic evolution and vulnerabilities of DTPs is a field of active research, and although we summarize key findings in the next part, many avenues remain to be elucidated.

A previous study has revealed that aldehyde dehydrogenase (ALDH) plays a pivotal role in maintaining drug-tolerant persistence by ameliorating elevated reactive oxygen species (ROS) levels and avoiding toxic effects. Pharmacologic inhibition of ALDH activity leads to the accumulation of toxic ROS, resulting in DNA damage and apoptosis, thereby eradicating DTPs [3]. In line with this finding, DTPs have been shown to depend on phospholipid glutathione peroxidase 4 (GPX4) for survival, which mitigates oxidative stress by reducing the lipid hydroperoxides. Consequently, inhibition of GPX4 has been demonstrated to induce ferroptosis in DTPs across various cancer types [4]. In addition to oxidative stress-related alterations, a series of metabolic remodeling linked to fatty acid oxidation (FAO) has been discovered in DTPs. A recent study by Shen et al. has identified that peroxisomal lipid metabolism represents an “Achilles’ heel” in BRAF-mutated melanoma persister cells. The peroxisomal FAO is upregulated in DTPs via mediating PPARα–ACOX1 axis and blockade of FAO either through knockdown of key peroxisomal FAO enzyme or treatment with FAO inhibitor thioridazine hampers the emergence of resistance to targeted therapy [5]. Similarly, another study has revealed that cycling and non-cycling persister cells exhibit distinct transcriptional and metabolic profiles, with a metabolic shift to FAO associated with the proliferative capacity of cycling persisters. Impeding such metabolic reprogramming reduces the proportion of cycling persisters and delays disease recurrence [6]. These studies underscore the critical link between oxidative stress and FAO in DTPs. Intriguingly, recent studies have unprecedentedly uncovered a striking resemblance between the DTP state and embryonic diapause, driven by developmentally conserved mechanisms. In these states, MYC and mTOR pathways suppress biosynthetic and metabolic activities at the transcriptional level [7]. At the same time, a more recent study has revealed the metabolic switch to FAO occurs in an *in vitro* diapause system, identifying FOXO1 and l-carnitine as key regulators [8]. These findings suggest that lipid metabolism plays a crucial role in DTPs formation, potentially representing a shared mechanism between DTPs and diapause states. Further research is warranted to explore this metabolic orchestration and to uncover deeper conceptual and metabolic connections between DTPs and diapause blastocysts.

Recently, a study has deciphered the dynamic trajectories of cell-state transitions during adaptions to anticancer therapies across temporal dimensions, employing a system-level longitudinal framework. This research identifies a progressive enhancement of cell fitness, termed the “resistance continuum,” which involves stepwise transcriptional, epigenetic, and metabolic reprogramming. In particular, the study utilizes a CRISPR-based metabolic gene-knockout library screen to reveal systems-level metabolic dependencies along this resistance continuum. These include alterations in nucleotide metabolism, the pentose phosphate pathway (PPP), cholesterol biosynthesis, and oxidative phosphorylation. Of note, the study finds that drug-induced ROS production activates the NRF2 antioxidant response, which mitigates ROS via modulation of glutathione metabolism and the PPP, while also supplying precursors for nucleotide biosynthesis. Additionally, hyperactivation of NRF2 increases reliance on glutaminolysis, highlighting a vulnerability to the glutaminase (GLS) inhibitor CB-839 that depletes glutamate, a critical component for both glutathione production and nucleotide biosynthesis via tricarboxylic acid cycle intermediates [9]. This study emphasizes the value of longitudinal assessments in understanding drug-tolerant states that emerge during cancer treatment, offering insights into potential therapeutic vulnerabilities along the resistance continuum.

Intriguingly, our work has provided a fascinating insight into the role of acetylcholine (ACh), a key neurotransmitter markedly accumulated in DTP cells, in regulating the formation of DTPs. The accumulation of ACh is induced by the upregulated expression of choline acetyltransferase (ChAT), the rate-limiting enzyme in ACh biosynthesis, via the mediation of YAP signaling. Importantly, both genetic and pharmacological manipulation of ACh biosynthesis or ACh signaling regulates DTP formation *in vitro* and *in vivo*. Strikingly, pharmacologically targeting ACh/M3R signaling axis with darifenacin, an FDA-approved drug, has been shown to retard tumor relapse *in vivo*. Mechanistically, the study highlights that upregulated ACh metabolism confers drug tolerance by activating the WNT signaling pathway via muscarinic receptor 3 (M3R). Furthermore, the aberrant ACh metabolism observed in patients with non-small cell lung cancer (NSCLC) correlates with the EGFR-TKI response rate and progression-free survival. These findings identify that targeting the ACh/M3R/WNT axis could offer a promising therapeutic approach to overcome drug tolerance to EGFR-TKI in NSCLC [10]. Beyond these findings, the extensive studies suggest a pivotal role for the nervous system and tumor-associated nerve networks in cancer progression and metastasis, mediated by neurotransmitter signaling. In the future, it will be worth exploring whether tumor-derived ACh impacts the immune microenvironment. The similar mechanisms bridging neuronal signaling and drug resistance in cancer could potentially pave the way to develop novel therapeutic interventions.

**Figure 1.**
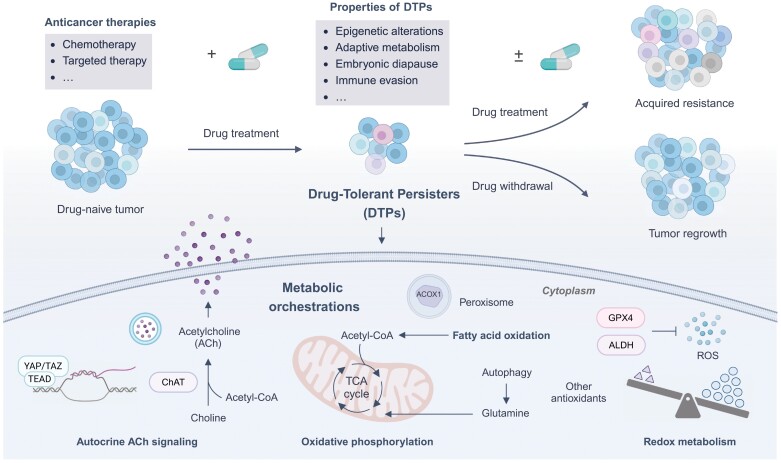
**Metabolic remodeling in drug-tolerant persister cells.**The schematic illustration unveils the key properties of drug-tolerant persister cells (DTPs), which specifically exhibits the metabolic orchestrations in the formation of DTPs.

In summary, these pioneering studies elegantly highlight the importance of metabolic orchestration in the formation of DTPs and subsequent tumor relapse (Fig. 1). Despite these advances, current research has predominantly focused on redox and energy metabolism, leaving a broader spectrum of reprogrammed metabolic pathways in DTPs underexplored. A more comprehensive map of these pathways, along with the discovery of previously unappreciated metabolic vulnerabilities, is essential for developing effective treatments. In addition, although tumor cell-intrinsic metabolic adaptions have been reported, the complex metabolic interactions between DTPs and tumor microenvironment remain largely unexplored. Moreover, DTPs exhibit remarkable metabolic heterogeneity, yet metabolomics at single-cell resolution for metabolic assessment presents profound challenges, making it difficult to fully capture this variability. Notably, the collection of longitudinal samples, both *in vitro* and *in vivo*, and a deeper understanding of metabolic trajectories across temporal and spatial dimensions during drug treatment are also key obstacles.

To tackle these challenges, a series of strategies must be pursued. The identification of more effective metabolic targets for DTPs eradication by integrating cutting-edge analytical techniques, including multi-omics approaches, single-cell analyses, and spatial and temporal omics, will be critical to further progress. Additionally, the development and refinement of experimental models that authentically capture the complex DTP state in clinical cancer patients is imperative. Moreover, fostering collaborations between researchers and clinicians will be essential for advancing this field. Specifically, the collection and interpretation of longitudinal samples from cancer patients will be crucial in unveiling the biology of persistence and drug resistance in real-world clinical settings. Ultimately, deepening our understanding of DTP biology will open avenues for developing novel therapeutic strategies that target these highly resilient cells, thereby inducing more durable clinical responses and improving patient outcomes.
